# *Peptidylarginine deiminase type 4* deficiency reduced arthritis severity in a glucose-6-phosphate isomerase-induced arthritis model

**DOI:** 10.1038/srep13041

**Published:** 2015-08-21

**Authors:** Yu Seri, Hirofumi Shoda, Akari Suzuki, Isao Matsumoto, Takayuki Sumida, Keishi Fujio, Kazuhiko Yamamoto

**Affiliations:** 1 Department of Allergy and Rheumatology, Graduate School of Medicine, the University of Tokyo, Bunkyo-ku, Tokyo, 113-8655, Japan; 2 Laboratory for Rheumatic Diseases, SNP Research Center, The Institute of Physical and Chemical Research (RIKEN), 1-7-22 Suehirocho, Tsurumi-ku, Yokohama City, Kanagawa 230-0045, Japan; 3 Division of Clinical Immunology, Major of Advanced Biomedical Applications, Graduate School of Comprehensive Human Sciences, Tsukuba University, Tsukuba, Ibaraki, 305-8575, Japan

## Abstract

Peptidyl arginine deiminase 4 (PAD4) is an enzyme that is involved in protein citrullination, and is a target for anti-citrullinated peptide antibodies (ACPAs) in rheumatoid arthritis (RA). Genetic polymorphisms in the *PADI4* gene encoding PAD4 are associated with RA susceptibility. We herein analyzed the roles of *PADI4* in inflammatory arthritis using a glucose-6-phosphate isomerase (GPI)-induced arthritis (GIA) model in *Padi4* knockout (KO) mice. Arthritis severity, serum anti-GPI antibody titers, and IL-6 concentrations were significantly reduced in *Padi4* KO mice. The frequency of Th17 cells was decreased in GPI-immunized *Padi4* KO mice, whereas WT and *Padi4*-deficient naïve CD4^+^ T cells displayed the same efficiencies for Th17 cell differentiation *in vitro*. In addition, the numbers of myeloid lineage cells were reduced with the increased expression of pro-apoptotic genes in GPI-immunized *Padi4* KO mice. Furthermore, the survival of *Padi4*-deficient neutrophils was impaired *in vitro.* Our results suggest that *PADI4* exacerbates arthritis with diverse immunological modifications.

Rheumatoid arthritis (RA) is characterized by sustained and destructive polyarthritis with an autoimmune background. Environmental and genetic factors have both been shown to contribute to the pathogenesis of RA; however, its pathogenesis has not yet been elucidated in detail^1^,^2^. A recent genome-wide associated study (GWAS) provided a larger amount of genetic information on RA^3^. *Peptidylarginine deiminase type 4* (*PADI4*) was the initially identified genetic susceptibility locus of RA in the non-major histocompatibility molecule (MHC) locus^4^. Furthermore, although most of the recently identified autoimmunity loci are shared among multiple autoimmune diseases, *PADI4* is uniquely associated with RA^5^, which suggests the principle importance of *PADI4* in the pathogenesis of RA.

*PADI* genes encode PAD proteins that convert arginine residues to citrulline in the presence of sufficient Ca^2+^ concentrations^6^, and protein citrullination is one of the post-translational modifications that have been reported. *PADI4* possesses some distinctive characteristics from the four other human *PADI* isotypes. It is mainly expressed in myeloid lineage cells, such as neutrophils and monocytes, and its expression is inducible under inflammatory conditions^6^,^7^. *PADI4* has nuclear localization signals that permit its translocation to the nucleus^8^. The RA-susceptible *PADI4* haplotype has been shown to give rise to more stable *PADI4* mRNA and is associated with increases in PAD4 protein levels^4^. These findings suggest that the enhanced and uncontrolled production of citrullinated antigens results in the development of an anti-citrullinated peptide antibody (ACPA) and the occurrence of joint inflammation in patients with the RA-susceptible *PADI4* haplotype. However, two recent reports demonstrated that *PADI4* is associated with ACPA-negative RA patients in Asian populations and one of the risk factors for bone destruction independent of ACPA status^9^,^10^. These findings suggest an ACPA-independent pathway for the association of *PADI4* with the pathogenesis of RA. Previous studies in which the homeostatic activities of *PADI4*, such as transcriptional modulation, cell cycle/apoptosis regulation, and the acquirement for pluripotency, were demonstrated to support this possibility^11^,^12^,^13^,^14^,^15^,^16^,^17^.

Recombinant human glucose-6-phosphate isomerase (rhGPI)-induced arthritis (GIA) is an established and immunologically characterized model of RA^18^. The development and exacerbation of GIA has been shown to depend on CD4^+^ T cells, especially Th17 cells and IL-6^18^,^19^. Therefore, GIA is a suitable model not only for examining arthritis, but also evaluating various immunological events that occur during the course of arthritis. We herein tested the GIA model in *Padi4* KO mice and demonstrated a reduction in joint inflammation. We observed decreases in the number of Th17 cells, levels of serum anti-GPI antibodies and IL-6, and the number of myeloid lineage cells in GPI-immunized *Padi4* KO mice. Furthermore, the survival of *Padi4*-deficient neutrophils was impaired *in vitro.* Taken together, *Padi4* exacerbated RA with diverse immunological modifications.

## Results

### Reduced severity of GIA in *Padi4* KO mice

**Figure 1:**
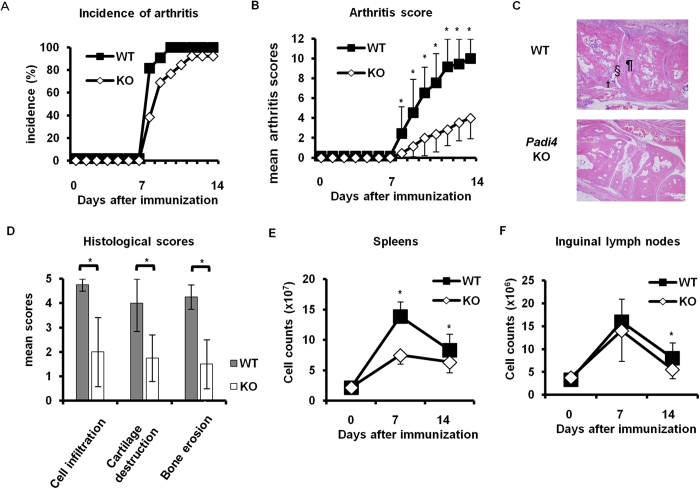
Comparative analyses of wild-type (WT) and *Padi4* knock out (KO) mice of recombinant human Glucose-6-phosphate isomerase (rhGPI)-induced arthritis (GIA). (**A**) The incidence of GIA. (**B**) The mean severity of GIA graded by previously reported methods. (WT n = 11, *Padi4* KO n = 13) (**C**,**D**) Representative arthritic joint sections and mean histological scores were graded by previously reported methods 14 days after the immunization. ¶: inflammatory cell infiltration, §: cartilage destruction, ^**†**^: bone erosion. Gray bars; WT, open bars; *Padi4* KO. (**E**,**F**) The numbers of splenocytes and inguinal lymph node cells were counted 0, 7, and 14 days after the immunization. Pre-immunized (WT n = 3, *Padi4* KO n = 3), 7 days after the immunization (WT n = 9, *Padi4* KO n = 9), 14 days after the immunization (WT n = 14, *Padi4* KO n = 16). *p < 0.05.

WT and *Padi4* KO mice both developed arthritis approximately 7 days after the rhGPI immunization and arthritis scores increased between 8 and 14 days after the immunization. Most of the immunized WT and *Padi4* KO mice developed arthritis, and no significant difference was observed in the incidence of arthritis (Fig. 1A). Arthritis severity scores were significantly lower in *Padi4* KO mice than in WT mice (Fig. 1B). The histological scores for inflamed joints were also lower in *Padi4* KO mice than in WT mice (Fig. 1C,D). These results demonstrated that *Padi4* was associated with the exacerbation of GIA.

We initially focused on the number of immune cells in lymphoid organs in the pre-arthritic phase (7 days after the immunization) and arthritic phase (14 days after the immunization). In the pre-arthritic phase, the increased number of whole splenocytes in number was attenuated more in *Padi4* KO mice than in WT mice (Fig. 1E). In the arthritic phase, the numbers of splenocytes and iLN cells were both lower in *Padi4* KO mice than in WT mice (Fig. 1E,F). These results suggested that the pre-arthritic immune responses in *Padi4* KO and WT mice differed.

### Lower serum anti-GPI antibody titers in *Padi4* KO GIA mice

**Figure 2:**
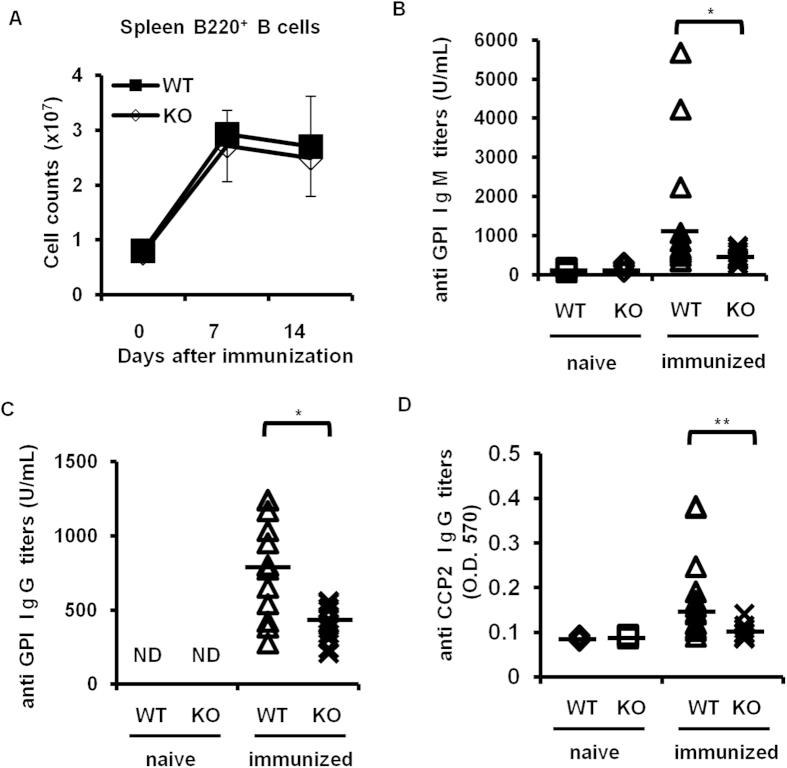
B cells and serum anti-GPI antibodies in *Padi4* KO mice after the GPI immunization. (**A**) The number of B220^+^ B cells in spleens was counted 0, 7, and 14 days after the immunization. Pre-immunized (WT n = 3, *Padi4* KO n = 3), 7 days after the immunization (WT n = 6, *Padi4* KO n = 6), 14 days after the immunization (WT n = 11, *Padi4* KO n = 13). (**B**–**D**) The titers of serum anti-GPI IgM, IgG antibodies and anti-CCP2 IgG antibodies were measured before and 14 days after the immunization. Pre-immunized (WT n = 3, *Padi4* KO n = 3), 14 days after the immunization (WT n = 13, *Padi4* KO n = 13), N.D.: not detected. *p < 0.05.

We then examined B cell subsets and anti-GPI antibody production following the rhGPI immunization because B cells and the anti-GPI antibody were required for the development of GIA^18^,^20^. After the rhGPI immunization, no significant difference was observed in the total number of B cells in the spleen or iLN cells between WT and *Padi4* KO mice (Fig. 2A). Regarding serum antibodies, although rhGPI-immunized WT mice developed significant amounts of anti-GPI IgM and IgG antibodies, *Padi4* KO mice only produced limited titers of anti-GPI IgM and IgG antibodies (Fig. 2B,C). Moreover, ACPA titers after rhGPI immunization were lower in *Padi4* KO mice (Fig. 2D).

### Decreased number of Th17 cells in *Padi4* KO GIA mice

**Figure 3:**
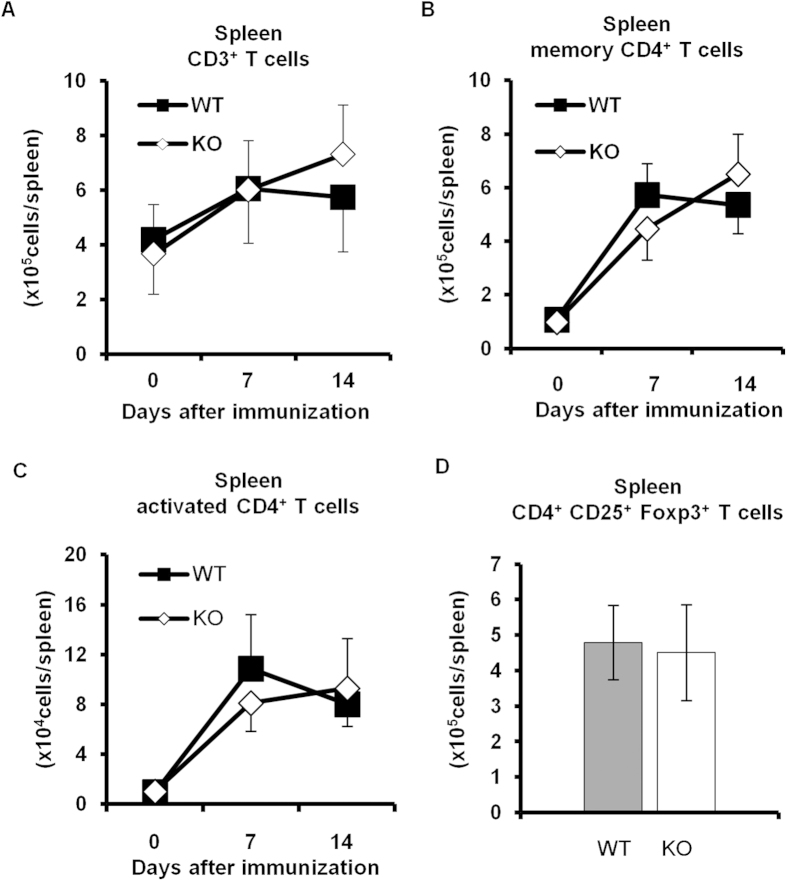
T cell proliferation and activation in *Padi4* KO mice after the GPI immunization. (**A–C**) The numbers of CD3^+^ T cells, CD4^+^ CD62L^−^ CD44^+^ memory T cells, and CD4^+^ CD62L^−^ CD69^+^ activated T cells in spleens were counted 0, 7, and 14 days after the immunization. Pre-immunized (WT n = 3, *Padi4* KO n = 3), 7 days after the immunization (WT n = 6, *Padi4* KO n = 6), 14 days after the immunization (WT n = 11, *Padi4* KO n = 13). (**D**) The number of CD4^+^ CD25^+^ Foxp3^+^ regulatory T cells in spleens was counted 14 days after the immunization (WT n = 8, *Padi4* KO n = 10). Gray bars; WT, open bars; *Padi4* KO.

**Figure 4:**
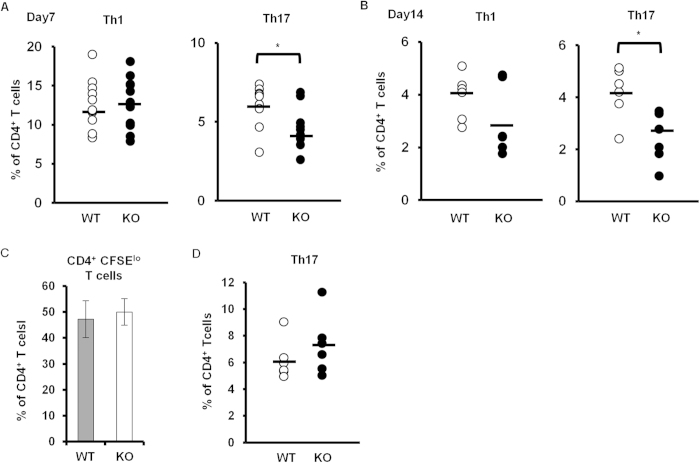
Th17 cell differentiation in *Padi4* KO mice after the GPI immunization. (**A**,**B**) Proportions of Th1 and Th17 cells in the total amount of CD4^+^ T cells from inguinal lymph nodes 7 and 14 days after the immunization. Seven days after the immunization (WT n = 9, *Padi4* KO n = 11), 14 days after the immunization (WT n = 6, *Padi4* KO n = 6). (**C**) The CFSE dilution of CD4^+^ T cells from GPI-immunized inguinal lymph nodes was measured in response to an *ex vivo* stimulation with GPI (WT n = 3, *Padi4* KO n = 3). (**D**) Proportion of *in vitro* differentiated Th17 cells in CD4^+^ T cells under Th17-polarizing conditions (WT n = 3, *Padi4* KO n = 3). *p < 0.05, ***p < 0.001.

CD4^+^ T cells, especially Th17 cells, play a pivotal role in the development of GIA^21^. In non-immunized DBA1 mice and GIA mice, the numbers of CD3^+^ T cells, CD62L^−^ CD44^+^ CD4^+^ memory T cells, and CD62L^−^ CD69^+^ CD4^+^ activated T cells in the spleens and iLNs were similar between *Padi4* KO and WT mice (Fig. 3A–C), as was the number of CD25^+^ Foxp3^+^ CD4^+^ regulatory T cells (Fig. 3D). However, the frequency of iLN GPI-specific Th17 cells, but not Th1 cells, was significantly reduced in *Padi4* KO mice either 7 or 14 days after the rhGPI immunization (Fig. 4A,B). On the other hand, no significant change was noted in the proliferation of CD4^+^ T cells, as analyzed by CFSE dilution, between WT and *Padi4* KO mice (Fig. 4C).

To determine whether the impairment observed in Th17 cell differentiation in *Padi4*-deficient mice was caused in a T cell intrinsic manner, naive WT or *Padi4*-deficient CD4^+^ T cells were cultured under Th17 cell-polarizing conditions. *Padi4*-deficient CD4^+^ T cells as well as WT CD4^+^ T cells efficiently differentiated into Th17 cells *in vitro* (Fig. 4D). These results suggested that the decreases observed in Th17 cells in *Padi4* KO GIA mice were due to some extrinsic causes during the course of the rhGPI immunization rather than to intrinsic defects in *Padi4*-deficient CD4^+^ T cells.

### Decreased serum IL-6 concentrations in *Padi4* KO GIA mice

**Figure 5:**
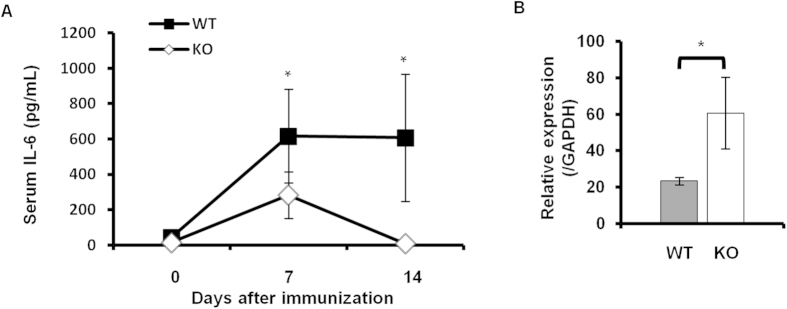
(**A**) Serum IL-6 concentrations in *Padi4* KO mice 7 and 14 days after the GPI immunization. Serum IL-6 concentrations were measured by an enzyme-linked immunosorbent assay (WT n = 6, *Padi4* KO n = 7). Gray dots; WT, open dots; *Padi4* KO. Comparisons were performed using two-way analysis of variance followed by Bonferroni *post-hoc* test. *p < 0.05. (**B**) IL-6 gene expression levels in spleens were measured by quantitative PCR 7 days after the immunization (WT n = 3, *Padi4* KO n = 3). Gray bars; WT, open bars; *Padi4* KO.

IL-6 is a key cytokine in the induction of Th17 cells, and is also important for joint inflammation. Serum IL-6 concentrations were already reduced in *Padi4* KO mice in the pre-arthritic phase, and the evident reduction continued in the arthritic phase (Fig. 5A). A previous study reported that myeloid lineage cells were the main source of IL-6 in a GIA model^19^. Although we investigated the expression of IL-6 in the spleen in the pre-arthritic phase, a significant decrease was not observed in *PADI4* KO mice (Fig. 5B).

### Decreased numbers of CD11b^**+**^ cells in *Padi4* KO mice after the rhGPI immunization

**Figure 6:**
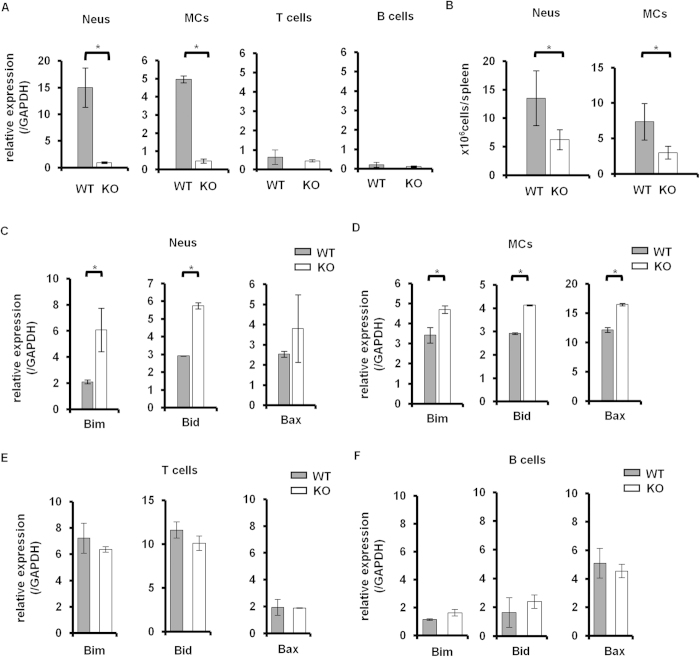
Neutrophil and monocyte numbers after the GPI immunization. (**A**) The expression of *Padi4* in CD11^+^ Ly-6G^+^ neutrophils (Neus), CD11^+^ Ly-6C^+^ Ly-6G^−^ monocytes (MCs), CD3^+^ T cells, and CD19^+^ B cells was measured by quantitative PCR 7 days after the immunization (WT = 3, *Padi4* KO = 3). (**B**) The numbers of Neus and MCs in spleens were counted 7 days after the immunization (WT n = 9, *Padi4* KO n = 9). (**C–F**) Pro-apoptotic gene expression in Neus, MCs, T cell, and B cells was measured by quantitative PCR 7 days after the immunization (WT n = 3, *Padi4* KO n = 3). Gray bars; WT, open bars; *Padi4* KO. *p < 0.05.

**Figure 7:**
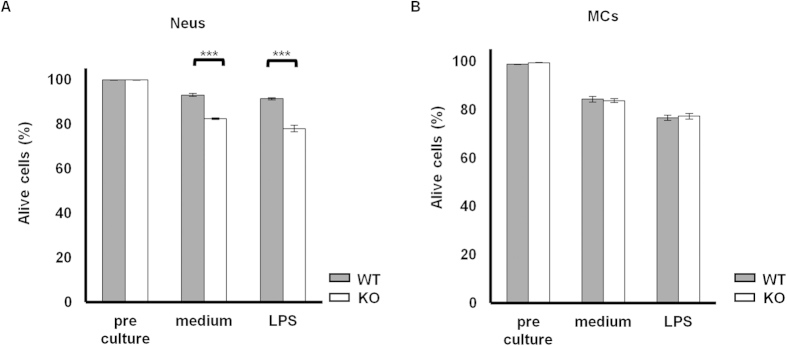
*In vitro* survival of *Padi4*-deficient neutrophils and monocytes. (**A,B**) Splenic CD11^+^ Ly-6G^+^ neutrophils (Neus) and CD11^+^ Ly-6C^+^ Ly-6G^−^ monocytes (MCs) were cultured with or without LPS and their survival was measured by FACS (WT n = 3, *Padi4* KO n = 3). Gray bars; WT, open bars; *Padi4* KO. *p < 0.05.

Previous studies demonstrated that the *Padi4* gene was mainly expressed in myeloid lineage cells^6^. In the case of the GIA model, *Padi4* was dominantly expressed in CD11b^+^ Ly-6G^+^ neutrophils (Neus) and CD11b^+^ Ly-6C^+^ Ly-6G^−^ monocytes (MCs), not T cells or B cells (Fig. 6A). As shown in Fig. 1D, although the total cell numbers of splenocytes and iLN cells were significantly decreased in *Padi4* KO GIA mice, the number of B cells or T cells remained unchanged in *Padi4* KO mice (Figs 2A and 3A). In contrast, the numbers of Neus and MCs were significantly decreased in the spleens of *Padi4* KO mice 7 days after the rhGPI immunization (Fig. 6B). Furthermore, the expression of pro-apoptotic genes, such as Bid, Bad, and Bax, was increased in *Padi4*-deficient Neus and MCs from rhGPI-immunized mice (Fig. 6C,D). The expressions of these pro-apoptotic genes were not different in the lymphoid lineage cells (Fig. 6E,F). Moreover, the survival of *PADI4*-deficient Neus, not MCs, was impaired in the *in vitro* culture with or without the LPS stimulation (Fig. 7). These results suggested that *PADI4* controls the survival of myeloid lineage cells.

## Discussion

In the present study, we demonstrated that the severity of arthritis was reduced in GPI-immunized *Padi4* KO mice by various changes in immune reactions, including myeloid lineage cells, IL-6, antibodies and Th17 cells. Previous studies reported the effects of a deficiency in or the inhibition of *Padi4* in inflammatory arthritis models. In the case of antibody-dependent arthritic models, *Padi4* was dispensable for the arthritis effector phase. Administration of the Pan - PAD molecule inhibitor, Cl-amidine, did not reduce the severity of anti-type II collagen antibody-induced arthritis (CAIA)^22^. In the KxB/N serum transfer arthritis model, no significant differences were found in the severity of arthritis in *Padi4* KO mice and WT mice^23^. On the other hand, in the type II collagen-induced arthritis (CIA) model, the administration of Cl-amidine reduced anti-type II collagen antibody production and the severity of arthritis^22^. Furthermore, *Padi4* KO TNF alpha transgenic (Tg) mice had milder arthritis and less activated CD4^+^ T cells and some ACPAs than WT–TNF alpha Tg mice^24^. Therefore, the characteristics and roles of ACPAs in mouse models of arthritis are complicated, and will be elucidated, although serum ACPA development was significantly supressed in *Padi4* KO GIA mice. Taken together, we demonstrated diverse immunological modifications, including helper T cell development, cytokine production, and immune cell apoptosis in *Padi4* KO mice, which played certain roles in the pathogenesis of arthritis.

Previous studies demonstrated that antibody production was impaired under *Padi4*-deficient conditions^22^,^24^. GPI-immunized *Padi4* KO mice had lower serum anti-GPI antibody titers than WT mice. However, in terms of arthritogenic potential, serum anti-GPI antibody titers did not correlate with the severity of arthritis in the GIA model^18^,^20^. Therefore, the reduction observed in arthritis severity in *Padi4* KO mice may be explained by factors other than reductions in anti-GPI antibody levels. In previous studies on GIA models, IL-6, IL-17, and CD4^+^ T cells were identified as crucial components for the development and severity of GIA^21^. In *Padi4* KO mice, serum IL-6 concentrations and the number of Th17 cells were significantly decreased. Therefore, there is a possibility that the decreased activation of the IL-6-Th17 axis, a direct arthritogenic cascade, reduced the severity of arthritis in the GIA models. However, it remains unclear whether the suppression of arthritis in *Padi4* KO GIA was relevant to Th17 and/or Th1-mediated inflammatory process, and further investigations are required in this point. With regard to IL-6, high serum concentration was reported to be a characteristic finding and play a pivotal role in newly onset ACPA-positive RA patients^25^. In our experiment, GIA mice similarly exhibited serum IL-6 elevation in the early phase in a *Padi4*-dependent manner. Therefore, *Padi4*-mediated control of IL-6 may contribute to the early stage of RA. Furthermore, CD11b^+^ cells have been identified as one of the major IL-6 sources in mice models of RA^19^,^26^. Since a decrease in the number of CD11b^+^ cells was evident in GPI-immunized *Padi4* KO mice, this shortage of IL-6 sources represents one possible explanation for the reduced arthritis severity in *Padi4* KO mice. The dominant infiltrating cells were polymorphonuclear neutrophils (PMNs) in WT GIA mice (Fig. 1C). Enhanced apoptosis of *Padi4*-deficient neutrophils may explain the reduced cellular infiltration in the joints of Padi4 KO GIA mice. Otherwise, decreased numbers of Th17 cells could regulate the chemoattraction of the neutrophils to the joints, because Th17 cells were reported to secrete CXCL8, which was associated with neutrophil chemotaxis^27^.

Neutrophils strongly express *Padi4* and play important roles not only in direct tissue injury, but also in the modulation of adaptive immune responses^28^. The expression of *Padi4* was previously reported to be crucial for the formation of neutrophil extracellular traps (NETs)^29^,^30^,^31^,^32^. NETs contain citrullinated proteins, such as citrullinated histone, and are assumed to be a source of autoantigens. NETs also exhibit an adjuvant activity that activates dendritic cell (DC) function and T cell priming^31^,^32^,^33^,^34^,^35^,^36^. Apart from being a possible source of IL-6 in the GIA model, CD11b^+^ neutrophils play pivotal roles in the maturation of adaptive immune responses and GIA development via the production of NETs. Although the precise roles of NETs in mice arthritis models, including GIA, remain unclear, some reports suggested the importance of NETs in human RA^33^,^34^. Especially, Khandpur *et al.* reported that NETs induce IL-6 and IL-8 secretion from RA fibroblast-like synoviocytes (FLSs)^34^. Therefore, loss of NETs could be associated with the decrease of serum IL-6 in *Padi4* KO GIA mice. Furthermore, the expression of *Padi4* may control the survival and functions of neutrophils. More detailed information is needed about the nature of *Padi4* in order to elucidate these points.

The molecular mechanisms of action of *Padi4* in CD11b^+^ cells remain unknown. PAD4 was previously shown to be located in the nucleus, and regulated gene transcription through the citrullination of histones^11^,^13^. An analysis of the relationship between *Padi4* and tumor cells has also provided some insights into this topic. Previous studies showed that PAD4 inhibited p53 and promoted cell proliferation by cancelling G_1_ arrest in the cell cycle^17^. PAD4 also displayed anti-apoptotic effects by suppressing several pro-apoptotic molecules^14^,^15^,^16^. Therefore, we speculated that nuclear molecular targets coupled with PAD4 can regulate gene transcription and cell survival.

In summary, we herein demonstrated reductions in the severities of GIA in *Padi4* KO mice. Moreover, diverse modifications in the immune responses were observed in GPI-immunized *Padi4* KO mice, such as decreases in serum IL-6 levels, Th17 cell development, and anti-GPI antibody production. Especially, the number of peripheral myeloid lineage cells was significantly decreased in GPI-immunized *Padi4* KO mice, and the survival of *Padi4*-deficient neutrophils was impaired *in vitro.* Taken together, *Padi4* exacerbate inflammatory arthritis with diverse immunological modifications which play certain roles in the pathogenesis of arthritis. Based on the reduction of the severities of arthritis in *Padi4* KO mice, *Padi4* suppression could be clinically adopted in human translational medicine.

## Materials and Methods

### Mice

Age- and Sex-matched DBA1J mice were supplied by SLC Japan (Shizuoka, Japan). *Padi4* knockout (*Padi4* KO) mice were generated by the deletion of exon 1 in the B6 background. The *Padi4* KO B6 mice were backcrossed to the DBA/1J background for at least 17 generations to obtain *Padi4* KO DBA1J mice with more than 99.5% identity with the original DBA1J mice^37^. The protocols used were approved by the Ethical Committee on Animal Experiments of the University of Tokyo, and all animal experiments were conducted in accordance with institutional and national guidelines.

### GIA

rhGPI was generated and purified as described previously^38^. DBA/1J male mice were immunized with 300 μg of rhGPI plus an equal volume of Complete Freund’s adjuvant (DIFCO LABORATORIES, Detroit, MI, USA). Mice were sacrificed and blood, spleen, inguinal lymph nodes, and limbs were gathered 7 or 14 days after the immunization. In the histological analysis, joint sections were prepared and stained with hematoxylin and eosin by the Biopathology Institute Company (Oita, Japan). Synovial tissues were graded by inflammatory cell infiltration, cartilage destruction, and bone erosion as described previously^39^.

### *CD4* ^**+**^ *T cell culture*

Inguinal lymph node (iLN) cells (1 × 10^5^/mL) were cultured with irradiated splenocytes (1 × 10^6^/mL) with or without rhGPI (5 μg/mL) in RPMI 1640 medium supplemented with 5% FCS, 2 mM L-glutamine, 100 U/ml penicillin, 100 μg/ml streptomycin, and 5 × 10^−5^ M 2-mercaptoethanol in a humidified incubator at 37 °C, 5% CO_2_, for 72 h. In some experiments, mouse CD4^+^ T cells were labeled with 1 × 10^−6^ M of carboxyfluorescein diacetate succinimidyl ester (CFSE) and then cultured on anti-CD3 antibody and anti-CD28 antibody-coated plates under Th17-inducing conditions^38^. To achieve intracellular cytokine staining, cells were stimulated with 1 × 10^−5^ M PMA and 0.5 μg/ml ionomycin and treated with Golgistop (BD Bioscience) 3 hours before being harvested.

### Flow cytometry

Cell surface markers and intracellular cytokines were analyzed and cell sorting was performed by FACS Vantage (BD Bioscience). Endogenous Fc receptors were blocked using an anti-mouse CD16/CD32 antibody (2.4.G2; BD Bioscience) and subsequently stained using fluorescein isothiocyanate, phycoerythrin, allophycocyanin (APC), APC-Cy7, and the following biotin-conjugated antibodies. Anti-mouse Ly-6G (1A8), Ly-6C (AL21), CD25 (PC61), NK1.1 (PK136), CD3 (17A2), CD8a (53-6.7), IL-17A (TC11-18H10), and Annexin V antibodies were obtained from BD Bioscience. Anti-mouse F4/80 (BM8), CD4 (GK1.5), CD11c (N418), CD45R/B220 (RA3-6B2), CD62L (MEL-14), CD44 (IM7), and CD69 (H1.2F3) antibodies were obtained from Biolegend (San Diego, CA, USA). Anti-mouse CD11b (M1/70) and IFN-gamma (XMG1.2) antibodies were obtained from eBioscience (San Diego, CA, USA). APC-Cy7-conjugated streptavidin was obtained from Biolegend. Intracellular cytokine staining was performed with Fixation/Permeabilization Concentrate and Diluent (eBioscience) following the manufacturer’s protocol.

### ELISA

Serum IL-6 concentrations were measured by Mouse IL-6 High Sensitivity ELISA according to the manufacturer’s protocol (eBioscience). Serum anti-GPI IgM and IgG antibodies were measured by ELISA according to a previously reported protocol with slight modifications^39^. A serum mixture of 4 WT mice 14 days after immunization was used as 1000 unit (U) standard of the anti-GPI antibody. Serum anti-CCP2 antibodies were measured by Mesacup CCP2 test (MBL) in accordance to the previous study^40^.

### RNA extraction and quantitative PCR

RNA was extracted from cells using the RNeasy Micro Kit (Qiagen, Valencia, CA, USA). Extracted RNA was reverse-transcribed to cDNA with random primers (Invitrogen, Carlsbad, CA, USA) and Superscript III according to the manufacturer’s protocol (Invitrogen). Quantitative PCR was performed using the SYBR Green Master Mix (Qiagen) and iCycler system (Bio-rad, Hercules, CA, USA). The results obtained were shown in terms of relative expression levels compared to GAPDH. The primers used in quantitative PCR were listed in Supplementary Table.

### *In vitro* survival assay

Isolated splenocytes from non-immunized mice were cultured in RPMI1640 medium supplemented with 5% FCS, 2 mM L-glutamine, 100 U/ml penicillin, 100 μg/ml streptomycin, and 5 × 10^−5^ M 2-mercaptoethanol in the presence or absence of LPS (100 ng/mL) (Sigma) in a humidified incubator at 37 °C, 5% CO_2_, for 24 h. The percentages of Annexin V-negative CD11b^+^ Ly6G^+^ and Ly6C^+^ cells were analyzed by FACS.

### Statistical analysis

Statistical analyses were performed by SPSS (IBM, Armonk, NY, USA). Data were expressed as the mean ± SEM. Differences were compared by the non-parametric test. Comparisons of more than three groups were performed using two-way analysis of variance followed by Bonferroni *post-hoc* test. *P* values less than 0.05 were considered significant.

## Additional Information

**How to cite this article**: Seri, Y. *et al.* *Peptidylarginine deiminase type 4* deficiency reduced arthritis severity in a glucose-6-phosphate isomerase-induced arthritis model. *Sci. Rep.* **5**, 13041; doi: 10.1038/srep13041 (2015).

## Supplementary Material

Supplementary Information
