# Supplementary material for: Peptidylarginine deiminase type 4 deficiency reduced arthritis severity in a glucose-6-phosphate isomerase-induced arthritis model
Source: Sci Rep. 2015 Aug 21;5:13041. doi: 10.1038/srep13041 (PMC4544002; doi:10.1038/srep13041)
Supplement: Supplementary Information [file srep13041-s1.pdf]

Supplementary information

Peptidylarginine deiminase type 4 deficiency reduced arthritis severity in a glucose-6-phosphate isomerase-induced arthritis model

Yu Seri, Hirofumi Shoda, Akari Suzuki, Isao Matsumoto, Takayuki Sumida, Keishi Fujio, and Kazuhiko Yamamoto

Supplementary Table

List of primers

	Forward	Reverse
Gapdh	TTCACCACCATGGAGAAGGC	GGCATGGACTGTGGTCATGA
Padi4	TGACCAATGGATGCAGGACG	CTCTGTCCCTCGGGGAGTC
Bax	TAGCAAACTGGTGCTCAAGG	TCTTGGATCCAGACAAGCAG
Bid	AGACGAGCTGCAGACAGATG	GGTCCATCTCATCGCCTATT
Bad	GAGGAGGAGCTTAGCCCTTT	AGGAACCCTCAAACTCATCG
IL6	GAGGATACCACTCCCAACAGACC	AAGTGCATCATCGTTGTTCATACA
